# Simplified Asset Indices to Measure Wealth and Equity in Health Programs: A Reliability and Validity Analysis Using Survey Data From 16 Countries

**DOI:** 10.9745/GHSP-D-15-00384

**Published:** 2016-03-25

**Authors:** Nirali M Chakraborty, Kenzo Fry, Rasika Behl, Kim Longfield

**Affiliations:** a Population Services International, Washington, DC, USA; b Independent consultant, London, UK; c University of California, Global Health Group, San Francisco, CA, USA

## Abstract

**Background:**

Social franchising programs in low- and middle-income countries have tried using the standard wealth index, based on the Demographic and Health Survey (DHS) questionnaire, in client exit interviews to assess clients’ relative wealth compared with the national wealth distribution to ensure equity in service delivery. The large number of survey questions required to capture the wealth index variables have proved cumbersome for programs.

**Methods:**

Using an adaptation of the Delphi method, we developed shortened wealth indices and in February 2015 consulted 15 stakeholders in equity measurement. Together, we selected the best of 5 alternative indices, accompanied by 2 measures of agreement (percent agreement and Cohen’s kappa statistic) comparing wealth quintile assignment in the new indices to the full DHS index. The panel agreed that reducing the number of assets was more important than standardization across countries because a short index would provide strong indication of client wealth and be easier to collect and use in the field. Additionally, the panel agreed that the simplified index should be highly correlated with the DHS for each country (kappa ≥ 0.75) for both national and urban-specific samples. We then revised indices for 16 countries and selected the minimum number of questions and question options required to achieve a kappa statistic ≥ 0.75 for both national and urban populations.

**Findings:**

After combining the 5 wealth quintiles into 3 groups, which the expert panel deemed more programmatically meaningful, reliability between the standard DHS wealth index and each of 3 simplified indices was high (median kappa = 0.81, 086, and 0.77, respectively, for index B that included only the common questions from the DHS VI questionnaire, index D that included the common questions plus country-specific questions, and index E that found the shortest list of common and country-specific questions that met the minimum reliability criteria of kappa ≥ 0.75). Index E was the simplified index of choice because it was reliable in national and urban contexts while requiring the fewest number of survey questions—6 to 18 per country compared with 25 to 47 in the original DHS wealth index (a 66% average reduction).

**Conclusion:**

Social franchise clinics and other types of service delivery programs that want to assess client wealth in relation to a national or urban population can do so with high reliability using a short questionnaire. Future uses of the simplified asset questionnaire include a mobile application for rapid data collection and analysis.

## Abstract (precis)

Many program implementers have difficulty collecting and analyzing data on program beneficiaries’ wealth because a large number of survey questions are required to construct the standard wealth index. We created country-specific measures of household wealth with as few as 6 questions that are highly reliable and valid in both urban and rural contexts.

## INTRODUCTION

The 2012 unanimous adoption of a United Nations resolution to promote universal health coverage has prioritized a global movement to ensure that all people obtain health services they need without suffering financial hardship.^1^ Despite the emphasis on government responsibility to provide primary health care, the private sector is still extensively used for health services in low- and middle-income countries (LMICs).^2^^,^^3^ Health expenditure in the private sector has been shown to account for 61% of the total health expenditure in low-income countries, and the majority of these costs are out of pocket, which can prove especially difficult for the poor.^4^^–^^6^ In spite of the financial hardship associated with accessing private-sector health services, particularly high-quality health services, clients often indicate a preference for the private sector because of perceived availability and customer service orientation.^2^^,^^6^^–^^8^ Interventions that harness the power of the private sector to increase the poor’s access to necessary, high-quality services without causing undue hardship have the potential to move countries closer to universal health coverage.

While working with the private sector offers great opportunity, it also comes with challenges. In most LMICs, there is no unified oversight of the private sector. Quality standards for private-sector service delivery are often lacking and when they do exist, there is little to no enforcement.^3^^,^^9^

In the mid-1990s, concerns over the quality of private-sector care led to the creation of social franchising—the application of commercial franchising concepts to deliver socially beneficial products and services in underserved communities worldwide.^10^ When applied to clinical care, social franchising connects a network of health care providers through formal agreements to deliver health services under a common franchise brand and to improve overall quality.^11^

The social franchising industry has grown from just a few clinical franchises in the mid-1990s to more than 90 franchises in 40 countries around the globe.^12^ Costs associated with starting and maintaining social franchises have historically been covered through large donor grants, and while social franchise programs can differ in their scale and scope of services offered, most have the common goal of serving the poor.^10^^,^^13^

To implement strategies that best reach the poor, social franchisors must first accurately capture the socioeconomic profile of the people they serve. This information allows them to understand if the right clients are benefiting from subsidized services and to subsequently make decisions about where to scale-up or modify programs to reach those most in need. This paper first describes the different approaches to measuring wealth and the way many social franchisors have tried to understand the wealth profile of their clients, and then proposes a simplified but robust methodology to improve programmatic understanding and use of wealth measurement.

To best reach the poor, social franchisors must first accurately capture the socioeconomic profile of the people they serve.

## APPROACHES TO MEASURING WEALTH

Although income may appear to be the most obvious indicator of wealth and is commonly used as a measure of economic status, it has been found to be extremely difficult to capture accurately. Economists have realized that income tends to fluctuate a great deal according to factors such as seasonality and migration, and it does not account for informal earnings, such as payments made in-kind. Furthermore, individuals are often reluctant to share information about their income openly, which makes it difficult to measure during household surveys.^14^^,^^15^ Thus, it is not the best practical measure of wealth to support programmatic decisions.

As a measure of wealth, income is extremely difficult to capture accurately.

One alternative has been to measure consumption instead of income. Economists believe that consumption data, representing the total value of household monetary expenditure and items received as gifts or produced by the household, can be both representative of longer-term wealth and less sensitive to fluctuations in income. This method is used extensively in the World Bank’s Living Standards Measurement Study surveys and national Household Income and Expenditure Surveys. However, the surveys are extremely lengthy and are impractical when the primary objective for health program implementers is to collect other information besides consumption data.^14^

In the late 1990s, Filmer and Pritchett discovered that household characteristics and material assets were much easier to capture and could be used as a proxy for consumption and, consequently, for economic status.^16^ This led to the creation of the wealth index. Data for the wealth index are usually collected through Demographic and Health Surveys (DHS) or other national surveys and cover household ownership of selected assets and quality of living standards, such as housing structure and access to utilities. The raw data are converted into a weighted index using principal components analysis, and populations are divided into quintiles of wealth, each representing 20% of the population.^15^ Quintile 1 represents the poorest segment of the population and quintile 5, the wealthiest. Other population-level indicators are then stratified by wealth, allowing for an understanding of equity. Equity refers to an absence of differences (in health indicators) that are avoidable, unfair, and unjust; in this paper, we focus on differences specifically related to socioeconomic status.^17^^–^^19^ The inclusion of these questions in all DHS and similar surveys has made the wealth index one of the most common measures of equity in health.^20^^,^^21^ All references to DHS in this article constitute a reference to any party engaged in the collection, analysis, and reporting of the publicly available DHS data and reports.

## CONVENTIONAL WAYS OF MEASURING EQUITY IN SOCIAL FRANCHISING

As a community of practice, social franchisors have identified several goals for social franchising and are working together to identify uniform metrics for each goal.^22^ One goal, equity, requires an understanding of the socioeconomic status of franchise clients. To identify a practical measure of equity that could be used to inform scale-up of social franchising or modifications to existing strategies, the Social Franchising Metrics Working Group—comprised of franchisors and their donors—has worked together to pilot and choose an appropriate measure. The working group started with a rigorous testing process in which both absolute and relative measures of wealth were piloted. Results from the pilot revealed that the wealth index most closely aligned with the needs of franchisors by providing results that were easier to interpret than other measures. To facilitate a common application of this procedure, the working group created data collection and analysis resources.

The wealth index, constructed by collecting data on asset ownership, is one of the most common measures of equity in health.

Data for the wealth index among social franchising clients are typically gathered through client exit surveys and then compared with the national wealth index generated from DHS data. The analytic methods, described elsewhere, have also been automated in a toolkit that franchisors can use.^21^^,^^23^ Despite the availability of data collection and analysis resources, large social franchising organizations such as Population Services International (PSI) have found it difficult to systematically and accurately collect wealth index data across its franchises, which in the case of PSI spans 27 countries. Reasons for difficulty are related both to survey implementation as well as replicability of analytic methods.

Gathering wealth index data is simpler than implementing traditional consumption surveys. However, the number of questions needed to capture the variables required for the wealth index, using DHS country-specific questionnaires, range from 25–50. This adds to survey length, particularly in an exit interview context, and can make data collection time consuming. Additionally, several required questions are difficult for data collectors to ask and for clients to answer, especially since exit interviews take place away from the household. Specifically, the following challenges have been identified as being too complicated for client surveys:

To capture the variables required for the wealth index, DHS surveys need to ask 25–50 questions.

- Respondents have difficulty estimating with confidence the number of hectares of agricultural land their household owns while away from the household.
- Respondents living in peri-urban or partially built-up rural areas are unable to confidently say whether their household is in an urban or a rural area. This is also difficult for data analysts to determine given that definitions of urban and rural residence vary by country.
- Questions on household characteristics are intended to be completed by trained interviewers observing the household. However, in a clinic setting, clients often find it difficult to correctly answer questions with long and detailed response-option lists. For example, the standard DHS question on the type of toilet in a household has 13 response options, some of which may seem similar to the respondent (e.g., ventilated improved pit latrines and pit latrines with slabs).

To improve transparency for data analysis and make the method accessible to all types of programs, a toolkit with standard syntax was created. The syntax mimicked the process used by the DHS Program in creating its country indices. Given variability in the DHS procedure, the toolkit’s syntax varies from country to country, including in response options, country-specific assets, and differences in treatment of livestock variables.

The challenges discovered in trying to apply a complex analytic method to data intended for program monitoring and improvement raised the question of whether a simpler index could be created. Simplifications, however, may result in less accurate wealth quintile assignment. In this article, we consider the practical advantages of various alternatives to the standard wealth index and assess the extent to which each alternative’s wealth quintile assignment agrees with that of the standard wealth index. As program implementers, our primary concern is that our proposed methods pass muster within a larger community engaged in the measurement and use of equity data.

## METHODS

### Preliminary Analyses

To arrive at an alternative, simplified measurement approach, we adapted the Delphi method.^24^^,^^25^ We prepared preliminary analyses, described below, and presented them to an invited group of experts, who assembled in Washington, DC, in February 2015 for a panel meeting. The panel was comprised of 15 collaborators (6 men, 9 women), representing a variety of stakeholders including donors, franchise program implementers, developers of the original wealth index methodology, and others working in public health programs actively engaged in the measurement of equity. Members of the panel were not considered human subjects but collaborators in the analysis. Data used in the analyses, described below, are publicly available and de-identified.

We used the most recent DHS, MIS (Malaria Indicator Survey), or AIS (AIDS Indicator Survey) data from 16 countries to assess the validity of each alternative wealth measure. MIS and AIS surveys are nationally representative, as are the DHS, and related resources are publicly available.^26^ The 16 countries were selected based on 2 main criteria:

- Implementation of a DHS VI survey, in which the original wealth index factor weights had been published on the DHS website^27^ by July 2015
- The presence of a known social franchise in operation.

We used national survey data from 16 countries to assess the validity of simplified asset indices against the standard DHS wealth index.

The 16 countries were: Bangladesh, Benin, Cambodia, Cameroon, Ethiopia, Malawi, Mozambique, Nepal, Nigeria, Pakistan, the Philippines, Rwanda, Senegal, Tanzania, Uganda, and Zimbabwe.

For each country, we compared 4 alternative wealth indices (described as A–D below) against the original DHS-calculated wealth index. The alternative indices had fewer variables than the original wealth index for each country. In these comparisons, the DHS wealth index was conceptualized as the “gold standard,” and we aimed to determine how reliable each alternative was against this standard.

Two quantitative measures were used:

- Percent agreement, to determine what percent of individuals were assigned to the same quintile in the alternative measure as they would have been assigned to in the original
- Cohen’s kappa statistic (k), to take into account agreement that could have happened by chance alone.

Percent agreement can range from 0 to 100, while kappa ranges from -1 to 1, where 0 indicates that all agreement is due to chance alone. Researchers have proposed 2 alternative interpretations of kappa as follows: k<0 = no agreement; 0–0.20 = poor; 0.21–0.40 = fair; 0.41–0.60 = moderate; 0.61–0.80 = substantial; 0.81–1.0 = almost perfect.^28^ The second alternative interpretation is that k>0.75 is considered excellent, as per Fleiss.^29^

The original DHS wealth index for each country includes a set of common variables found in the DHS VI questionnaire, as well as country-specific variables.^30^ The original index routinely includes a measure of land size (number of hectares of land owned), whether the household is urban or rural, and number of animals owned, by animal type (cow, goat, chicken, etc.). Wealth indices are created for urban and rural respondents separately and then combined.^31^

**FIGURE 1.**
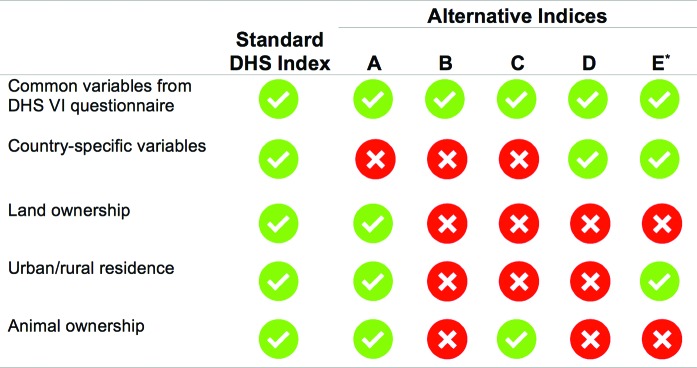
Types Included in the Standard DHS Wealth Index and in Each Alternative Simplified Index Abbreviation: DHS, Demographic and Health Survey. * Alternative E may contain some common questions from the DHS VI questionnaire and/or some country-specific variables. See main body of the article for further details.

To create each alternative index, we used a standardized process, beginning with the common variables in the DHS VI questionnaire.^23^ First, we recoded all categorical variables to binary variables. For questions with multiple response options (such as type of floor), we recoded each response option as a binary variable (none were merged together). Animal ownership was not recoded to a binary variable if it was entered as a continuous variable for each type of animal owned. We manually removed response options with zero cases, as well as those common variables that were not included in the country-specific questionnaire. We manually included country-specific variables. We then conducted a principal component analysis on all variables, with responses weighted at the individual level, and created a score from the factor weights of the first principal component. Scores were ordered and respondents were divided into 5 equal quintiles. Analyses were conducted using SPSS version 23. Figure 1 indicates which variables are present in each alternative asset index:

- Alternative A included all common variables that should be present in all DHS VI datasets, including land area and animal ownership. Wealth indices were created for urban and rural respondents separately and then combined. Country-specific assets were excluded.
- Alternative B included all common variables in the DHS VI questionnaire, except land area and animal ownership. It excluded country-specific assets, and a separate urban/rural analysis was not conducted.
- Alternative C excluded country-specific assets, land area, and the urban/rural analysis, but included animal ownership.
- Alternative D included country-specific assets. It excluded land area, animal ownership, and the urban/rural analysis.

### Consultation With Expert Panel

We presented the 4 alternatives to our panel. Panel members agreed that a shortened index is needed and that achieving the simplest and most practical questionnaires possible in each country was more important than standardizing questions across countries. All panel participants felt that a simplified approach would allow more programs to measure equity, resulting in better decision making and more equitable service delivery. It would also reduce the burden on clients being interviewed.

The panel made several recommendations, which necessitated the creation of another alternative (alternative E). First, the panel advised us to group the respondents into 3 groups. These groups, in order to be relevant to program decision making, were not terciles but rather the lowest 2 quintiles, the middle quintile, and the highest 2 quintiles, representing the relatively poor, middle, and rich. The panel felt that these 3 groupings would have greater face validity than the distinction between clients in the highest and second highest quintile, or between the lowest and second lowest quintiles. The panel also felt that presenting national quintiles alone would provide insufficient information for franchisors located primarily or solely in urban areas. They advised that the simplified set of questions should allow for sub-analyses on residence to determine the distribution of clients across urban wealth quintiles, with sufficient reliability. Further details about the variables included in alternative E are presented in the next section.

The best index of the 5 alternative options would be based upon 2 measures of agreement (percent agreement and Cohen’s kappa statistic of at least 0.75) and by comparing wealth quintile assignment in the new index to the full DHS wealth index. The same agreement rule applied to the urban sub-analysis: the simplified wealth index calculated among the urban respondents of the DHS survey should agree with the original wealth index in the urban stratum with a kappa statistic of at least 0.75.

### Revised Analyses for the Panel-Recommended Approach

To create the simplified wealth index for the new alternative E, we used an iterative process. We began by removing the variables related to land size (hectares) and animal ownership. Then, for each remaining variable, we created a measure of its importance to the overall wealth index by multiplying the absolute value of the factor weight of the first principal component (drawn from DHS documentation) by the standard deviation of that variable. Variables that have larger absolute factor weights explain a greater proportion of the variation in the construct. Multiplying the factor weight by the standard deviation captures variation in ownership within the population, and the overall procedure values variables with high variation. All included variables were binary, so the standard deviations were comparable in their units. To further simplify the index, we looked at each response option within a categorical variable independently. The goal was to create one list of variables that was sufficiently reliable in both the overall population and the urban population.

Specifically, we followed these steps to create the asset index for alternative E:

- Binary variables, including those constructed from categorical variables, from the original DHS wealth index were listed. These variables were ranked in order of their importance to national wealth index scores and separately ranked in order of importance to the urban wealth index.
- New wealth index scores were calculated for respondents in the DHS using the 5 most important variables in the overall and urban listings. Thus, up to 10 variables were included in the new wealth index calculation.
- Respondents were separated into wealth quintiles using the new scores. Respondents in urban areas were also assigned to urban-specific wealth quintiles.
- A cross-tabulation of the bottom 2 quintiles, middle quintile, and top 2 quintiles according to the original wealth index and according to the simplified wealth index was conducted. This allowed the calculation of the percentage of clients assigned to the same quintiles by the original DHS wealth index and the reduced set of variables, along with calculation of the kappa statistic. This was done for both the national wealth quintiles and for the urban wealth quintiles.
- Steps 2–4 were repeated until the smallest number of variables were found that met the reliability criteria of kappa ≥ 0.75 for both the national and the urban samples.
  - In cases where the kappa statistic for either the urban or national index or both were below 0.75, the next variable in the list from the distribution with the lower agreement was added, and steps 2–4 were repeated. This process was repeated until both the urban and national indices had kappa statistics of 0.75 or greater.
  - In cases where both urban and national indices had a kappa statistic above 0.75, the smallest list of variables was generated. Variables were removed in ascending order of importance, from the distribution with the lower kappa statistic. Steps 2–4 were repeated, until removing further variables resulted in a kappa statistic below 0.75.

## RESULTS

Table 1 presents the number of survey questions required to calculate the standard DHS wealth index as well as each of the 5 alternative indices presented to the expert panel. Note that the number of survey questions required to calculate the original wealth index for each country is not equal to the number of variables required in analysis, as categorical responses are converted into binary variables. Each alternative index contained fewer questions than the original DHS survey. The number of questions required for alternative E was fewer than for any of the other alternatives, for all countries presented. The average reduction in questions for index E from the original DHS survey was 66%, ranging from an 85% decrease in Benin (41 to 6 questions) to a 32% decrease in Malawi (25 to 17 questions).

The average number of questions required to construct the simplified index E was reduced by 66% from the original DHS wealth index.

**TABLE 1:** Number of Survey Questions Required for the Standard DHS Wealth Index and for Each Alternative Simplified Index

Country, Survey Type and Year	Variables Included^a^	Number of Survey Questions					
	Standard DHS Index	Standard DHS Index	Alternative A	Alternative B	Alternative C	Alternative D	Alternative E
Bangladesh, DHS 2011	CS, U/R, H, A	33	25	17	22	30	8
Benin, DHS 2011–12	CS, U/R, H, A	41	31	21	28	30	6
Cambodia, DHS 2010	CS, H	30	29	21	26	26	14
Cameroon, DHS 2011	CS, U/R, H, A	47	31	21	28	30	9
Ethiopia, DHS 2011	CS, U/R, H, A	36	29	20	26	25	14
Malawi, MIS 2012	U/R, H, A	25	26	19	23	19	17
Mozambique, DHS 2011	U/R, H, A	33	29	21	26	21	10
Nepal, DHS 2011	CS, U/R, H, A	44	30	20	27	29	10
Nigeria, DHS 2013	CS, U/R, H, A	26	30	21	27	28	11
Pakistan, DHS 2012–13	CS, U/R, H, A	47	29	21	26	34	14
Philippines, DHS 2013	CS, U/R	30	19	19	19	25	9
Rwanda, MIS 2013	CS, U/R, H, A	38	28	19	25	19	15
Senegal, DHS 2012-13	CS, U/R, H, A	41	28	19	25	28	18
Tanzania, AIS 2012–13	CS, U/R, H, A	37	31	21	28	24	8
Uganda, DHS 2011	CS, U/R, H, A	41	30	20	27	28	10
Zimbabwe, DHS 2010–11	CS, U/R, H, A	38	28	21	25	26	16

Abbreviations: AIS, AIDS Indicator Survey; DHS, Demographic and Health Survey; MIS, Malaria Indicator Survey.

a Variables included in the original DHS analysis, beyond the core variables from the DHS VI questionnaire. CS, country-specific; U/R, urban and rural areas analyzed separately before combining; H, Hectares or land area; A, animals.

In Table 2, we present 2 measures of reliability, the percent agreement and kappa statistic, between the original wealth index and each alternative index A–D. The reliability calculated here compares respondent movement between each of the 5 quintiles. The percent agreement and kappa statistic were highest overall for alternative D (median agreement, 83.26%; median kappa, 0.79). Although alternative B had fewer question types than alternative C, it produced a higher median agreement and kappa statistic (median agreement, 77.90% vs. 76.10%, respectively; median kappa, 0.72 vs. 0.70, respectively).

**TABLE 2:** Reliability Between the Standard DHS Wealth Index and Each Alternative Index Included in the Preliminary Analysis

	Alternative A		Alternative B		Alternative C		Alternative D	
Country	Agreement	Kappa	Agreement	Kappa	Agreement	Kappa	Agreement	Kappa
Bangladesh	73.20%	0.665	68.10%	0.601	60.00%	0.500	83.22%	0.790
Benin	85.40%	0.818	83.30%	0.791	83.90%	0.799	83.30%	0.791
Cambodia	73.20%	0.665	78.90%	0.736	75.20%	0.690	85.26%	0.816
Cameroon	81.10%	0.764	78.60%	0.733	79.10%	0.738	84.40%	0.805
Ethiopia	65.90%	0.574	62.10%	0.526	62.60%	0.533	69.30%	0.616
Malawi	88.60%	0.857	73.10%	0.664	64.80%	0.560	73.10%	0.664
Mozambique	87.90%	0.848	78.70%	0.734	74.50%	0.681	78.70%	0.734
Nepal	74.60%	0.682	77.60%	0.720	77.20%	0.714	87.55%	0.844
Nigeria	76.40%	0.705	72.20%	0.652	77.00%	0.712	74.86%	0.686
Pakistan	74.20%	0.678	73.50%	0.669	71.80%	0.648	89.22%	0.865
Philippines	79.20%	0.740	78.40%	0.730	78.40%	0.730	88.17%	0.854
Rwanda	88.80%	0.860	78.20%	0.728	71.40%	0.642	78.20%	0.728
Senegal	80.90%	0.761	76.40%	0.705	80.50%	0.754	77.45%	0.718
Tanzania	81.50%	0.769	80.30%	0.753	75.10%	0.689	85.34%	0.817
Uganda	76.50%	0.708	72.60%	0.657	69.00%	0.612	86.92%	0.837
Zimbabwe	86.50%	0.832	81.70%	0.772	78.70%	0.734	80.73%	0.759
**Median**	**80.05%**	**0.751**	**77.90%**	**0.724**	**76.10%**	**0.701**	**83.26%**	**0.791**
**Range**	**65.90% to 88.80%**	**0.574 to 0.860**	**62.10% to 83.30%**	**0.526 to 0.791**	**60.00% to 71.80%**	**0.500 to 0.648**	**69.30% to 89.20%**	**0.616 to 0.865**

Abbreviation: DHS, Demographic and Health Survey.

The subsequent analyses conducted as per panel group recommendations are presented in Table 3 (national) and Table 4 (urban only). The panel wished to compare alternatives B and D to the newly created alternative E, having decided that alternatives A and C were overly prone to respondent error due to the inclusion of questions on the number of animals owned. The comparisons are presented after combining the 5 wealth quintiles into 3 groups, which may be more programmatically meaningful. Consequently, the agreement and kappa statistics for alternatives B and D are greater in Table 3 than in Table 2, where respondent movement between quintile 1 and 2 would indicate error.

**TABLE 3:** Reliability^a^ of the National Wealth Distribution Between the Standard DHS Wealth Index and Each of 3 Alternative Indices Recommended by the Expert Panel Group

	Alternative B		Alternative D		Alternative E	
Country	Agreement	Kappa	Agreement	Kappa	Agreement	Kappa
Bangladesh	82.50%	0.727	90.74%	0.855	84.17%	0.753
Benin	91.02%	0.860	91.02%	0.860	84.91%	0.764
Cambodia	87.66%	0.807	92.23%	0.879	88.36%	0.818
Cameroon	88.20%	0.816	90.99%	0.859	85.26%	0.770
Ethiopia	75.97%	0.624	80.95%	0.702	84.73%	0.761
Malawi	83.44%	0.741	83.44%	0.741	84.30%	0.755
Mozambique	89.75%	0.840	89.75%	0.840	86.09%	0.783
Nepal	87.13%	0.799	94.08%	0.907	86.44%	0.788
Nigeria	87.52%	0.805	89.21%	0.831	85.22%	0.768
Pakistan	85.99%	0.781	94.47%	0.914	86.29%	0.786
Philippines	87.55%	0.805	93.09%	0.892	85.09%	0.766
Rwanda	86.83%	0.794	86.83%	0.794	84.13%	0.752
Senegal	87.92%	0.811	88.22%	0.816	86.22%	0.785
Tanzania	88.93%	0.827	92.12%	0.877	84.65%	0.756
Uganda	83.84%	0.748	92.88%	0.889	85.70%	0.777
Zimbabwe	91.48%	0.867	90.79%	0.856	85.46%	0.773
**Median**	**87.53%**	**0.805**	**90.89%**	**0.856**	**85.24%**	**0.769**
**Range**	**75.97% to 91.48%**	**0.624 to 0.867**	**80.95% to 94.47%**	**0.702 to 0.914**	**84.13% to 88.36%**	**0.752 to 0.818**

a Reliability assessed after grouping the 5 wealth quintiles into 3 groups: the lowest 40% (Q1+Q2), middle 20% (Q3), and richest 40% (Q4+Q5).

**TABLE 4:** Reliability^a^ of the Urban Wealth Distribution Between the Standard DHS Wealth Index and Each of 3 Alternative Indices Recommended by the Expert Panel Group

	Urban B		Urban D		Urban E	
Country	Agreement	Kappa	Agreement	Kappa	Agreement	Kappa
Bangladesh	83.69%	0.745	91.75%	0.871	85.00%	0.766
Benin	90.18%	0.847	91.91%	0.874	85.11%	0.768
Cambodia	86.34%	0.787	90.76%	0.856	85.37%	0.771
Cameroon	82.82%	0.732	86.15%	0.827	85.69%	0.776
Ethiopia	86.93%	0.796	88.10%	0.814	85.39%	0.771
Malawi	87.42%	0.803	87.42%	0.803	93.96%	0.906
Mozambique	94.54%	0.915	94.54%	0.915	89.01%	0.828
Nepal	87.26%	0.801	92.95%	0.890	84.26%	0.754
Nigeria	84.57%	0.759	89.43%	0.835	84.26%	0.754
Pakistan	78.41%	0.663	94.17%	0.909	84.27%	0.754
Philippines	84.39%	0.756	92.51%	0.883	85.24%	0.769
Rwanda	94.86%	0.920	94.86%	0.920	93.62%	0.900
Senegal	81.86%	0.717	86.79%	0.794	85.30%	0.770
Tanzania	91.01%	0.860	92.33%	0.880	83.95%	0.750
Uganda	81.55%	0.712	93.22%	0.894	84.12%	0.752
Zimbabwe	42.14%	0.593	75.02%	0.610	83.96%	0.750
**Median**	**85.46%**	**0.773**	**91.83%**	**0.873**	**85.17%**	**0.769**
**Range**	**42.14% to 94.54%**	**0.593 to 0.920**	**75.02% to 94.86%**	**0.610 to 0.920**	**83.95% to 94.00%**	**0.750 to 0.906**

a Reliability assessed after grouping the 5 wealth quintiles into 3 groups: the lowest 40% (Q1+Q2), middle 20% (Q3), and richest 40% (Q4+Q5).

The 6 questions chosen for Benin in alternative E, for example, produced a wealth distribution that agrees with the original wealth index 85% of the time among the national population, when the population was grouped into 3 meaningful divisions (Table 3). Despite having fewer questions, alternative E produced a higher kappa statistic in the national distribution than alternative B (including only the DHS core questions) for Bangladesh, Cambodia, Ethiopia, Malawi, Pakistan, and Uganda. Alternative E also produced a higher kappa statistic in the national distribution than alternative D (also including country-specific assets and animal ownership) for Ethiopia and Malawi. Similarly, when looking at the urban-specific distributions (Table 4), alternative E fared better than B in Bangladesh, Cameroon, Malawi, Pakistan, the Philippines, Senegal, Uganda, and Zimbabwe, and better than D in Malawi and Zimbabwe. In Zimbabwe, the effect of choosing variables that are strong predictors of wealth for the urban population was very evident—alternative E was the only one that produced a highly reliable result (k = 0.75 for alternative E; k<0.75 for alternatives B and D).

**FIGURE 2.**
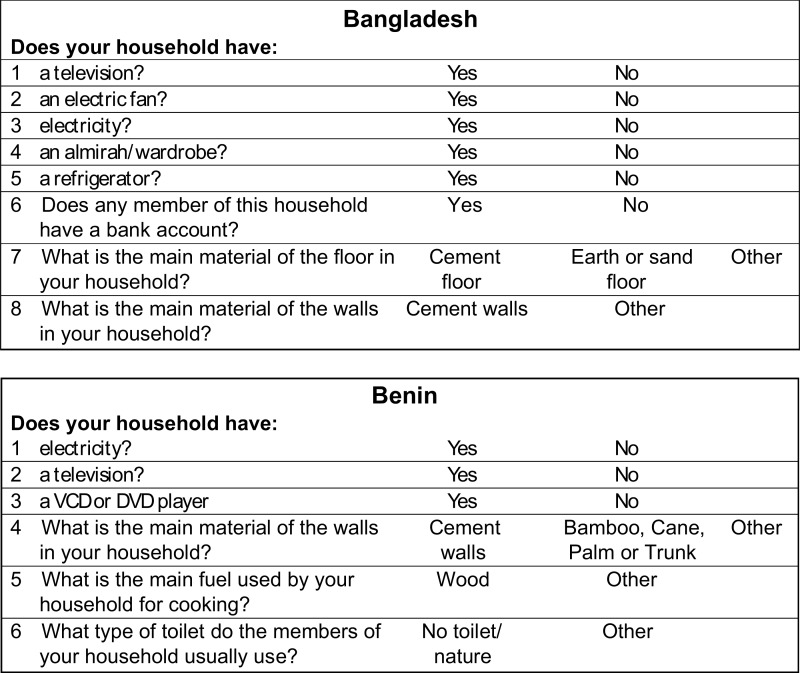
Example Simplified Wealth Asset Questionnaires for Alternative E for Bangladesh and Benin

Figure 2 shows the shortened alternative E questionnaires for Bangladesh and Benin (in English). In Benin, it is obvious, even without seeing the factor scores, that some questions are geared toward assessing wealth (e.g., having a DVD player) while others would be strong indicators of poverty (e.g., not having any toilet facility).

## DISCUSSION

This paper describes a methodological innovation that simplifies the collection of data to create relative measures of wealth within program populations. The premise behind the simplification process is that the DHS wealth index (both the construction and the resulting distribution) represents the “gold standard” for program implementers who need to understand the socioeconomic profile of their clients and beneficiaries. Thus, each alternative was judged against the gold standard, to determine if a sufficiently reliable alternative was possible.

Alternative E, presented here as the chosen, simplified approach because it required the fewest number of survey questions while maintaining a high enough reliability score, is a promising start for program implementers. Rather than advocating for a reduction in the DHS surveys, we acknowledge that quintiles from the standard DHS wealth index are a popular way to stratify populations, and program beneficiaries should be similarly categorized. The shorter questionnaires, however, are faster to use and therefore may improve the use of equity as a metric for internal decision making, as well as further its use as one for external accountability—a vision of the Social Franchising Metrics Working Group.

Index E was the simplified index of choice because it required the fewest number of survey questions while maintaining reliability in national and urban contexts.

Other concise approaches to assessing poverty status exist. The Grameen Foundation’s Progress out of Poverty Index (PPI) is limited to 10 questions for all available countries and is derived from a household income and expenditure survey.^32^ Thus, it is using the 10 easily answerable questions as a proxy of expenditure. It offers the user a probability that the respondent is above or below various poverty lines, thus measuring absolute poverty. A primary selling point of the PPI is the ability to compute the outcome by hand, as all of the scores are whole numbers. As with the approach we present, the questions for each country differ. The absolute measure from the PPI was previously piloted by franchise organizations, but it did not meet their diversity of needs and it was found to be more difficult for program decision makers to interpret than the wealth index.

Rutstein and Staveteig created one unique comparative wealth index, in which all countries with DHS data are benchmarked against Vietnam in 2002.^33^ In this measure, the items used to calculate the index are identical across countries, and while the measure is not relative within the country, it is still a measure of wealth relative to Vietnam. The comparative wealth index, however, was not considered to be a viable alternative by members of the expert group, including by the comparative index creators themselves.

We find it an advantage that our method is reliable for both national and urban populations. Previous pilot testing of wealth, benchmarked to the national population, as a metric of equity has indicated that franchised programs are not always able to act upon the results. Many franchise programs are primarily urban and peri-urban. When results have indicated that their clients are from the wealthiest 40% of the population, they have questioned the specificity of the measure and indicated they would like to see how their clients compared with others in the immediate area covered by the franchise network.^34^^,^^35^ Using the same short list of questions to compute equity in an urban sub-population will allow them this increased contextual information.

It is possible to include further sub-groups beyond the urban population in one of two ways. First, our iterative approach could be replicated to explore whether a shortened list of questions could be found that accurately divides members of the sub-group. A different set of questions than those described here may result. Second, the same short list of questions could be used, but the reference population changed when producing results. In this case, the results may not be as valid (kappa may not be greater than 0.75), but the results would be tailored to the sub-group of interest. One can imagine a large number of different short questionnaires or sub-group analyses that are possible. However, limiting the reference populations to the national and urban populations ensures practicality and comparability. With different evaluations using the same reference population, the level of poverty indicated by wealth quintiles is kept standard. These results are also comparable with data presented in DHS reports using national and urban quintiles. Interpretation of results should keep in mind that national or urban quintiles may not accurately represent quintiles specific to the sub-group eligible for the intervention in question.

### Limitations

We identify 3 primary limitations of our approach. First, the DHS surveys, a publicly available data source, are not available in all countries, nor do the surveys occur very frequently. The effect of the age of the source data on the inference to a current population remains to be assessed. Ownership of some assets, such as mobile phones, has rapidly increased in low-income countries. This can bias the results from a current survey, if, in an older reference population such as Cambodia in 2010, mobile phones were still indicative of being relatively wealthy but are now more pervasive. Second, following the DHS methodology, analysis is weighted to be generalizable to the whole population. However, in practice, exit surveys would be applied to a more narrow target group, such as women of reproductive age. If the target population is not evenly distributed across the 5 quintiles, this may introduce error into the results. Lastly, in shortening the questionnaires, we may have reduced our ability to distinguish between 2 adjacent quintiles. With fewer questions, it may not be possible to easily distinguish between quintiles 1 and 2, as the distribution is “lumpier.” For this reason, we grouped the respondents into 3 groups, which seemed more programmatically relevant, when assessing the reliability of the reduced survey. Piloting reduced questionnaires in varied settings may provide insight into whether less variability affects the utility of the findings.

## CONCLUSION

It is possible to use a shorter questionnaire to assess relative wealth within a sample that is benchmarked to the national population, and the resultant measure remains highly correlated to the original DHS wealth index. Through the engagement of an expert panel, this research has galvanized a great deal of interest among a variety of franchising programs, many of which are asking for shorter questionnaires to include within other surveys they conduct, such as for client satisfaction, as well as interest from the International Finance Corporation to assess the wealth of their project beneficiaries. The simplified asset questionnaires will also be embedded into a mobile application to make the collection and analysis of these data easier. (The shortened form of all questionnaires can be found online at www.equitytool.org.) The agreement of our expert panel—a seasoned group of methodologists, program implementers and donors—adds validity to the proposed methodology. Their conclusion that a simplified approach to assessing wealth is acceptable for programmatic decision making will benefit the use of this measure.

As current and former researchers within organizations implementing social franchising, the authors are keenly aware that the measurement of equity, in whatever form, is both desired by, and loathed by, their colleagues. International development organizations exist to serve the underserved, and this measure of socioeconomic status is only one way to define and measure underserved individuals. In many of the countries in which we work, most people are poor in absolute terms. A wealth index, as we have proposed, is relative, and compares those in the same country or sub-population with each other. The interpretation of results from this simplified method is context-specific and dependent upon program goals and needs of the eligible population. Future refinements should concentrate on providing both absolute and relative poverty information, in order to improve the understanding of the measure (for example, someone in the wealthiest quintile in Madagascar may still live on less than US$1.25/day) and the justification for providing subsidized services to individuals who appear wealthy on a relative scale.

## Notes

**Peer Reviewed**
